# Decline of olfactory function in Parkinson's disease: A ten-year longitudinal study

**DOI:** 10.1177/1877718X241298708

**Published:** 2024-12-08

**Authors:** Dareia S Roos, Martin Klein, Richard L Doty, Henk W Berendse

**Affiliations:** 1 Department of Neurology, Amsterdam UMC location Vrije Universiteit Amsterdam, Amsterdam, The Netherlands; 2 Amsterdam Neuroscience, Neurodegeneration, Amsterdam, The Netherlands; 3 Department of Medical Psychology, Amsterdam UMC location Vrije Universiteit Amsterdam, Amsterdam, The Netherlands; 4 Smell and Taste Center, Perelman School of Medicine, University of Pennsylvania, Philadelphia, PA, USA

**Keywords:** Parkinson disease, smell, disease progression, cognition

## Abstract

**Background:**

Olfactory dysfunction is a prodromal sign of Parkinson's disease (PD) present in up to 90% of patients. However, it is unclear whether or not olfactory function worsens over the course of the disease.

**Objective:**

In this study we examined whether the rate of decline of olfactory function in PD patients exceeds the expected age-related decline.

**Methods:**

Olfactory function was tested in 90 PD patients at baseline (age at baseline 58.3 years, 68.9% males) and an average 10 years later using the 40-item University of Pennsylvania Smell Identification Test (UPSIT). To screen for concomitant cognitive deficits as a potential confounder, the Mini-Mental State Examination (MMSE) was used.

**Results:**

At baseline, the mean UPSIT score was 22 points. Over the average 10-year follow-up period olfactory function decreased in 81.1% of PD patients, even in the youngest patients in whom no age-related decline was expected. The mean decrease was six UPSIT points (*p *< 0.001), which exceeds the expected age-related decline derived from a previous study. When excluding patients with an MMSE score below 24, reflecting cognitive deficits that might interfere with olfactory test performance, UPSIT score still decreased by almost 7 points over the follow-up period.

**Conclusions:**

Olfactory function in PD declines more rapidly with increasing disease duration than can be explained by aging or cognitive decline alone. As such, olfactory function appears to be a clinical marker of disease progression in PD that can be measured non-invasively and deserves consideration as part of multimodal phenotyping to monitor disease progression.

## Plain language summary

Up to nine out of ten patients with Parkinson's disease (PD) experience olfactory loss. We wanted to find out if this loss further declines with disease progression. We tested the sense of smell in 90 PD patients at the start of study and again about ten years later using the University of Pennsylvania Smell Identification Test (UPSIT). Patients were around 58 years old at study entry and most were men. We also tested their cognitive functioning to make sure it did not affect the smell test results. We found that the sense of smell got worse in eight out of ten PD patients over the ten years of follow-up. Also this was found in younger patients who wouldn’t normally lose that much of their sense of smell due to aging. On average, the smell test scores dropped by six points (out of a total of 40 points), which is more than expected in normal aging. Even after excluding patients with cognitive problems, the drop in UPSIT score was still about seven points. This shows that the sense of smell worsens faster in people with PD than would be expected from aging or cognitive decline alone. Therefore, testing olfactory function could be a useful way to monitor the progression of PD in a non-invasive way.

## Introduction

A reduced sense of smell is one of the most frequent and earliest non-motor symptoms in Parkinson's disease (PD). While hyposmia can be detected five to ten years prior to the first motor symptoms,^1,2^ it is still unknown whether and, if so, to what extent olfactory function declines with disease progression.

Most cross-sectional studies on olfactory function in PD patients, using either the University of Pennsylvania Smell Identification Test (UPSIT)^3^ or the Sniffin’ Sticks smell screening test,^4^ found no evidence for a time-dependent decline.^5–9^ However, two studies reported a negative correlation between disease duration and UPSIT scores.^10,11^ One study reported a similar correlation between disease duration and scores on the Sniffin’ Sticks odor discrimination, but not odor identification, test.^12^ Recently, in a cross-sectional study in 295 PD patients, we found hyposmia to be associated with various motor and non-motor symptoms, including cognitive deficits, depression, anxiety, autonomic dysfunction, and sleep disturbances.^13^

To date, only a limited number of PD studies have examined olfactory function longitudinally over the course of the disease.^14^ In the extant studies, follow-up periods were relatively short, various olfactory tests were employed, and sample sizes were typically small. No significant decrease in UPSIT scores over a two-year period was found in one study of 24 PD patients.^15^ In another study, a small, yet similar, decrease in UPSIT scores was noted in both 25 PD patients and 24 healthy controls over the course of a 45-month follow-up period.^16^ In three small-scale longitudinal studies using Sniffin’ Sticks, olfactory function of PD patients changed in an unpredictable way; some patients improved whereas others worsened.^17–19^ Taken together, previous cross-sectional and longitudinal studies failed to establish whether PD-related olfactory dysfunction is a stable disease feature or worsens with increasing disease duration.

Finding an answer to this question is first of all important from a pathophysiological point of view. In pre-motor stages of PD, alpha-synuclein pathology is present in the anterior olfactory nucleus, the olfactory bulb, and the lower brainstem. In later stages, such pathology spreads to ultimately involve large parts of the cerebral cortex.^20^ Given the early involvement of the olfactory bulb, it has been suggested that the causative agent of PD might enter the brain via the olfactory mucosa, potentially leading to a stable olfactory loss (one corollary of the ‘olfactory vector hypothesis’).^21^ A progressive deficit in olfactory function would indicate that the olfactory loss in PD reflects the ongoing accumulation of synuclein pathology in the brain.

Finding a progressive deficit in a quantitative test of olfactory function, could also be of value in monitoring disease progression and the assessment of the efficacy of disease modifying treatments.^22^ Unlike most other motor and non-motor symptoms of PD, olfactory tests are not responsive to dopamine.^15^ Hence, olfactory testing might be a unique measure of disease progression not confounded by dopamine-related drug therapies. Moreover, olfactory function can be easily measured with non-invasive low-cost practical psychophysical tests. That being said, the well-established age-related decline present in about a quarter of healthy middle-aged persons must be taken into account when examining longitudinal changes in the olfactory function of PD patients.^23–25^

The aim of the current study was to establish whether olfactory function in PD patients worsens with increasing disease duration, and to determine whether the rate of decline exceeds the known age-related reduction in the sense of smell. To address this aim, we measured olfactory function twice in a large a number of patients over an unprecedented follow-up period of ten years.

## Methods

### Participants

**Figure 1.**
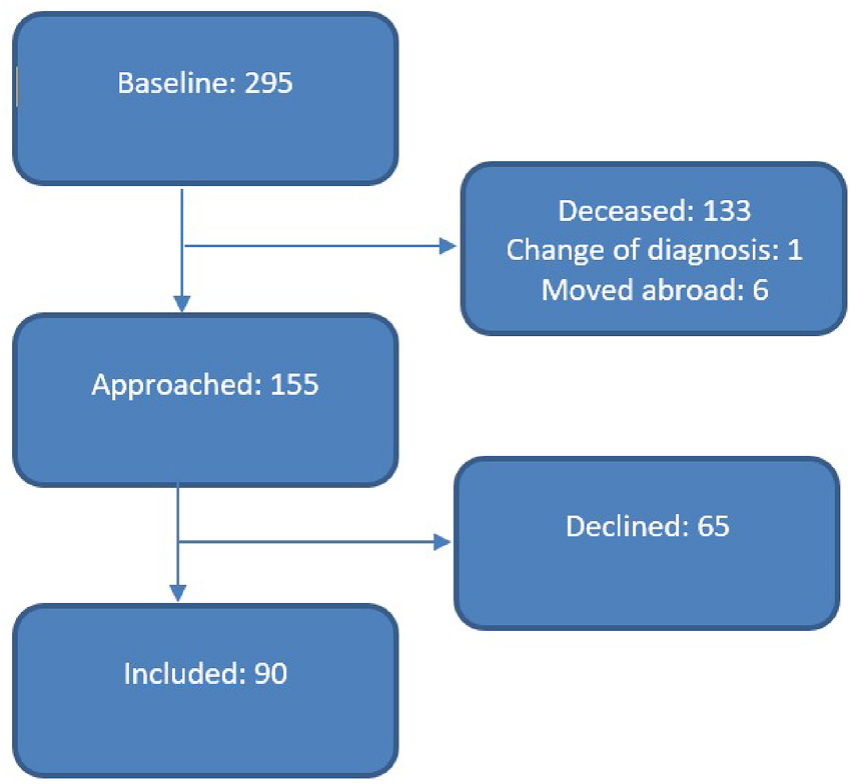
Patient selection for follow-up.

As described previously,^13^ 295 patients were recruited from the outpatient clinic for movement disorders at the Amsterdam UMC location Vrije Universiteit Amsterdam, between May 2008 and February 2014. Data were collected as part of routine clinical care. Ninety patients from the baseline cohort of 295 were included in the longitudinal study, with the remainder being either deceased (45%; n = 133), unwilling to participate (22%; n = 65), or otherwise lost to follow up (2.4%; n = 7) (Figure 1). Table 1 shows baseline characteristics of the patients included in the follow-up study compared to the patients lost to follow up or who declined participation.

**Table 1.** Patient characteristics at baseline for patients included in the follow-up study compared to the participants that declined to participate or were lost to follow-up.

	Patients included in study (n = 90)	Patients not included in study (n = 205)	Sign. (*p*)
Age, mean (SD), y	58.3 (9.4)	68.3 (9.2)	<0.001*
Males % (n)	68.9 (62)	57.1 (117)	0.070
Disease duration, mean (SD), y	4.5 (5.2)	5.6 (4.9)	0.005*
LEDD, mean (SD), mg	227.4 (352.4)	362.6 (482.5)	0.011*
UPSIT score, mean (SD)	22.2 (7.6)	19.5 (7.1)	0.003*
MMSE score, mean (SD)	28.6 (1.5)	26.7 (3.7)	<0.001*

LEDD: levodopa equivalent daily dosage; MMSE: Mini-Mental State Examination.

The patients fulfilled the UK Parkinson's Disease Brain Bank criteria for the clinical diagnosis of Parkinson's disease both at baseline and at follow-up, as determined by a neurologist specialized in movement disorders.^26^ Follow-up visits were performed between August 2020 and June 2021, either at the patients’ homes or the outpatient clinic, depending on their preference. This study was approved by the Medical Ethical Committee of Amsterdam UMC location Vrije Universiteit Amsterdam (VUmc; 2020.012). All patients gave written informed consent.

Disease duration was defined as the time from the self-reported onset of the first motor symptoms. The total dose of dopaminergic medication was converted to a levodopa equivalent daily dose (LEDD) using the following conversion rate: 100 mg levodopa equaling 133.33 mg levodopa with controlled release, 1 mg pramipexol (as salt), 5 mg ropinirol, 3.3 mg rotigotine, and 100 mg amantadine. Additionally, 10% was added to the total levodopa dose in case of the use of selegiline, rasagiline or safinamide, while 20% was added to the total levodopa dose in case of the use of a catechol-O-methyl transferase (COMT)-inhibitor.^27^ Demographic characteristics were collected, including the presence of deep brain stimulation (DBS), smoking habits, and the presence of active acute or chronic rhinosinusitis.

### Olfactory function

Olfactory function was measured with the Dutch version of the UPSIT.^3^ The UPSIT is a self-administered forced-choice odor identification test consisting of four test booklets, with ten items each (total score 40). The participant scratches the patch containing microcapsules filled with odorant, and then sniffs the released odor to choose one out of four options. The total score was calculated by adding up all correct answers. At baseline, patients completed the UPSIT at home; when responses to one or more items were missing, patients were asked to complete these items during their visit in the hospital. At follow-up the UPSIT was directly supervised. In rare cases when this could not occur, the average of non-missing values was used to impute up to two missing values.

The expected age-related decline in UPSIT scores was derived from an earlier cohort study performed by one of the authors, in which almost 1450 healthy controls were examined.^3^

### Cognitive screening

As a measure of overall cognitive function, the Mini-Mental State Examination (MMSE) was used at baseline and follow-up.^28^ The maximum score is 30 points, with higher scores corresponding to a better cognitive performance. A score below 24 was taken as indicative of cognitive deficits that might interfere with performing the UPSIT.^29^

### Statistical analysis

The data were analyzed using SPSS 28.0 (SPSS Inc., Chicago, IL, USA). Statistical significance was set at *p *< 0.05. Baseline demographic characteristics of PD patients included in the follow-up study and patients that declined participation were compared using an independent t-test, Mann-Whitney U test, or Chi-square test, depending on the data distribution. A paired-samples *t*-test was used to compare the UPSIT scores at baseline and follow-up. We used the Wilcoxon signed-rank test to compare MMSE scores at baseline and follow-up. McNemar's test was used for non-numerical data.

## Results

### Clinical characteristics

At baseline, mean age of the 90 PD patients that participated in the follow-up was 58 years. Mean disease duration, defined as the time from the self-reported onset of motor symptoms, was 4.5 years at baseline, and 14.2 years at follow-up. The levodopa equivalent daily dose increased by an average of 758 mg over ten years. As expected, patients showed disease progression over a ten-year period on the Hoehn & Yahr stage (Table 2). Additionally, the number of patients treated with Deep Brain Stimulation (DBS) increased (Table 2).

**Table 2.** Characteristics of the study population group.

	Baseline (n = 90)	Follow-up (n = 90)
Age, mean (SD), y	58.3 (9.4)	68.0 (9.4)
Males % (n)	68.9 (62)	
Disease duration, mean (SD), y	4.5 (5.2)	14.2 (5.4)
LEDD mean (SD), mg	227.4 (352.4)	985.4 (504.0)
DBS % (n)	1.1 (1)	26.7 (24)
Smoking status % (n)		
Never	52.2 (47)	52.2 (47)
Former	35.6 (32)	44.4 (40)
Current	12.2 (11)	3.3 (3)
H&Y stage % (n)		
Stage 1	18.9 (17)	-
Stage 1.5	13.3 (12)	-
Stage 2	51.1 (46)	32.2 (29)
Stage 2.5	11.1 (10)	30.0 (27)
Stage 3	5.6 (5)	26.7 (24)
Stage 4	-	10.0 (9)
Stage 5	-	1.1 (1)

LEDD: levodopa equivalent daily dosage; DBS: deep brain stimulation; H&Y: Hoehn & Yahr stage.

### Olfactory function

**Figure 2.**
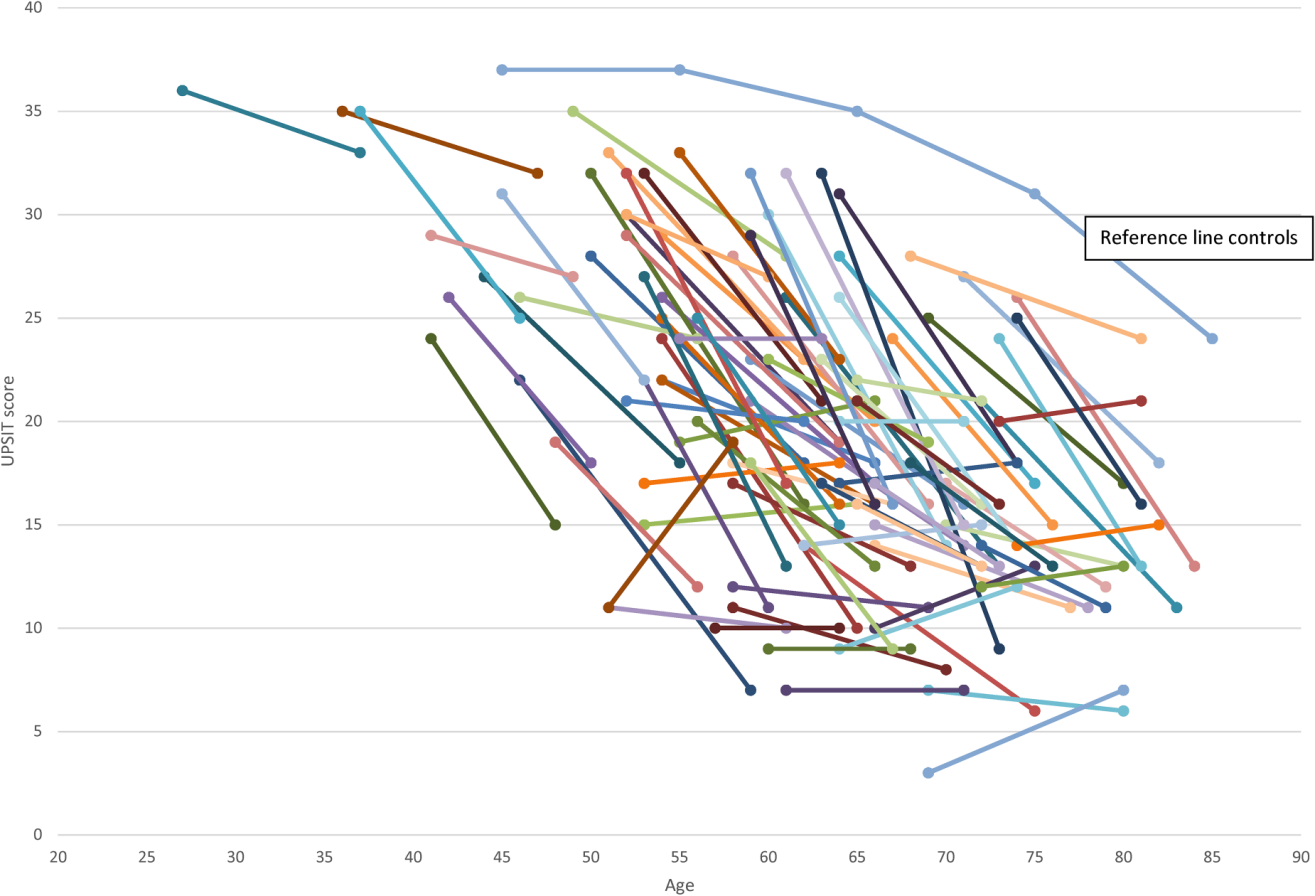
Spaghetti plot illustrating the change of UPSIT scores between baseline and follow-up for patients with PD with reference line for controls adapted from a study by Doty et al.^3^

Olfactory function declined by an average of 6.2 points from a mean of 22.2 points at baseline to 16.0 points at follow-up (Table 3). More than 81% (n = 73) of patients had a decrease in their UPSIT score, in 5.6% (n = 5) the score was unchanged, and in 13.3% (n = 12) it increased. Eleven patients showed improvement of one to four points, one single patient showed improvement of 8 points. A spaghetti plot shows the changes in UPSIT score for each individual participant (Figure 2). Baseline olfactory function was better in females compared to males; and declined more in females (Table 3). The expected age-related decline of olfactory function in healthy controls, derived from a study by Doty et al.,^3^ was added to the plot (Figure 2).

**Table 3.** UPSIT score, (chronic) sinusitis, and MMSE score at baseline and follow-up.

	Baseline (n = 90)	Follow-up (n = 90)	Sign. (*p*)
UPSIT score, mean (SD)	22.2 (7.6)	16.0 (5.5)	*p *< 0.001*
UPSIT score, mean (SD) - males (n = 62)	21.4 (7.5)	15.7 (5.5)	*p *< 0.001*
UPSIT score, mean (SD) - females (n = 28)	24.0 (7.8)	16.6 (5.4)	*p *< 0.001*
UPSIT score, mean (SD), excluding patients with (chronic) sinusitis (n = 82)	21.9 (7.6)	15.9 (5.5)	*p *< 0.001*
UPSIT score, mean (SD), excluding patients with MMSE <24 points and missing MMSEs	23.4 (6.8) (n = 76)	16.6 (5.2) (n = 76)	*p *< 0.001*
MMSE score, mean (SD)	28.6 (1.5) (n = 88)	27.1 (3.6) (n = 87)	*p *< 0.001**
MMSE score <24, % (n)	2.3 (2)	10.3 (9)	*p *= 0.016***

MMSE: Mini-Mental State Examination; *paired-samples *t*-test; **Wilcoxon signed-rank test; ***McNemar test.

At baseline, 12% of patients smoked, compared to 3% at follow-up. Six patients stopped smoking four months to 6 years prior to the follow-up visit. Two more patients stopped in the months directly prior to the follow-up visit. In all eight patients that quit smoking, UPSIT scores nevertheless decreased.

Eight patients reported having chronic sinusitis. When excluding these patients, olfactory function still decreased by six points (Table 3).

### Cognitive function

Two patients at baseline and three patients at follow-up refused to perform the MMSE. The mean score of the MMSE remained high at baseline and follow-up, and showed a statistically significant mean decline of 1.5 points (Table 3), but of minor clinical significance. At baseline, two patients had an MMSE score below 24. At follow-up, this had increased to nine patients. When excluding these 14 patients, the UPSIT score still decreased by 6.8 points over the follow-up period.

## Discussion

In the present study we observed that olfactory function in a sizable sample of PD patients worsened over a 10-year period by an average of six points as measured using the UPSIT. This exceeds by far the decline usually found in healthy subjects of similar age during a period of ten years.^3,7,23,25^ Olfactory test scores decreased in more than 80% of our PD patients, even in the youngest patients in whom no age-related decline was expected. The high rate of decline in the sense of smell was maintained after excluding patients with evidence of a cognitive deficit at follow-up. Also when excluding patients with (chronic) sinusitis, olfactory function decreased six points during follow-up.

Our findings contrast with those from earlier longitudinal studies, all of which reported no changes in olfactory test scores over a shorter follow-up period (i.e., ≤4.5 years).^15–19^ Although the majority of cross-sectional studies report no association between olfactory function and disease duration,^5–9^ in a number of studies negative correlations have been reported between these measures using the UPSIT^10,11,13^ or Sniffin’ Sticks.^12^ A likely explanation for the differences between our findings and the earlier longitudinal studies is our relatively large sample and our longer follow-up period of 10 years duration.

The pathophysiology of the PD-related changes we observed over time is enigmatic. It is possible that the initial olfactory loss is related to neuropathological changes in the olfactory nucleus and bulb at early disease stages.^20^ Worsening of olfactory function, as demonstrated in the present study, might simply reflect progression of these localized changes, as also supported by previous neuropathological findings.^30^ Further decline of olfactory function in later stages of PD also could be the result of alpha-synuclein pathology spreading to brain regions involved in higher order olfactory processing, such as the medial prefrontal cortex, insula, and orbitofrontal cortex.^31,32^ Although olfactory testing might be influenced by disease-related cognitive dysfunction, earlier research using the Picture Identification Test (PIT) suggests this is unlikely. Scores on the PIT, a cognitive test that assesses picture recognition of the same stimulus items used in the UPSIT,^33^ were found to be largely independent of the UPSIT scores of PD patients.^34,35^ Moreover, when excluding PD patients in the present study with an MMSE score below 24, a common cut-off point for dementia,^36^ olfactory function still decreased by the same degree. This suggests that cognitive functioning alone cannot explain the decline in olfactory function.

Although the age-related changes expected to occur over the time period of this study are less than the changes observed in the PD patients, some of the processes involved in producing age-related changes might nevertheless be accentuated in PD. These include not only age-related alterations in brain pathology as neuronal expression of aberrant neurodegenerative proteins and changes in neurotransmitter and neuromodulator systems, but also in nasal tissue engorgement which influences nasal airflow patterns, decrements in mucosal metabolizing enzymes, ossification of cribriform plate foramina, and loss of selectivity of receptor cells to odorants.^23,37^

In the current study, we have demonstrated that a progressive olfactory deficit occurs in PD that can be assessed using an easy-to-administer, noninvasive test. As disease-modifying treatments for PD are being developed, there is a need for monitoring biomarkers to measure treatment effects on disease progression. Ideally, such biomarkers should not be influenced by dopaminergic medications, cognitive deficits, or motor functioning,^15,38^ and should be non-invasive, practical, rapid, and inexpensive. In non-demented PD patients, olfactory testing seems to meet all these conditions, and would therefore seem to be an interesting element of multimodal clinical phenotyping to monitor disease progression.^39^

The most important strength of our study is the unprecedented long follow-up of ten years over which olfactory function was assessed in a large group of PD patients. Furthermore, all follow-up data was collected by the same researcher (D.R.), which increases the consistency of the collected data. A potential weakness is the fact that the patients were not supervised when filling in the UPSIT at the baseline study visit. If anything, however, this might have led to lower baseline scores, and thus have led to an underestimation of the actual difference between baseline and follow-up test scores. This could also explain why in some PD patients the total UPSIT score increased at the follow-up visit. That being said, other studies have not found scores based upon self-administration of the UPSIT to differ from those in which the administration was assisted by a technician.^40,41^ Another potential weakness is the relatively high number of participants that were lost to follow-up, largely due to deceased patients. As the patients lost to follow-up were older, had a longer disease duration, higher LEDD, lower UPSIT score and MMSE score, this may have resulted in a degree of attrition or survival bias. However, any bias towards patients with a more benign disease course would have resulted in an underestimate of the actual rate of decline in olfactory function. At the time we started our study, it was not yet known that COVID-19 can also cause long-term olfactory loss.^42^ Therefore, we cannot exclude the possibility that we included some asymptomatic individuals in whom olfactory function had decreased after a COVID-19 infection. However, this would not explain the pattern of decline of olfactory function in almost all participants as illustrated in Figure 2. Lastly, the lack of a control group is regrettable. At baseline, data was collected as part of routine clinical care; so no control group was available. However, the use of the age-related decline derived from a previous study in a very large number of healthy controls^3^ is a reliable alternative.

To conclude, olfactory function in PD declines more rapidly with disease duration than can be explained by aging or cognitive decline alone. As such, olfactory function appears to be a clinical marker of disease progression in PD that can be measured non-invasively and deserves consideration as part of multimodal phenotyping to monitor disease progression.
